# Supplementary material for: Self-supporting sulfur cathodes enabled by two-dimensional carbon yolk-shell nanosheets for high-energy-density lithium-sulfur batteries
Source: Nat Commun. 2017 Sep 7;8:482. doi: 10.1038/s41467-017-00575-8 (PMC5589916; doi:10.1038/s41467-017-00575-8)
Supplement: Supplementary file 2 — Supplementary Information [file 41467_2017_575_MOESM2_ESM.pdf]

Description of Supplementary Files

File name: Supplementary Information
Description: Supplementary figures, supplementary tables and supplementary references.

File name: Peer Review File

Supplementary Information

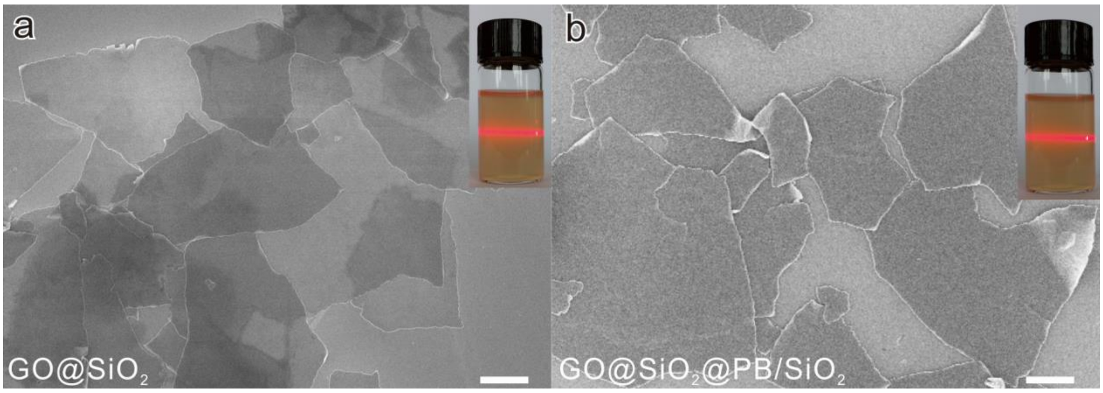

Supplementary Figure 1. SEM images of the intermediates. (**a**) GO@SiO_2; (**b**) GO@SiO_2@PB/SiO_2, insets: optical images of the corresponding dispersions. Scale bars, 1 μm.

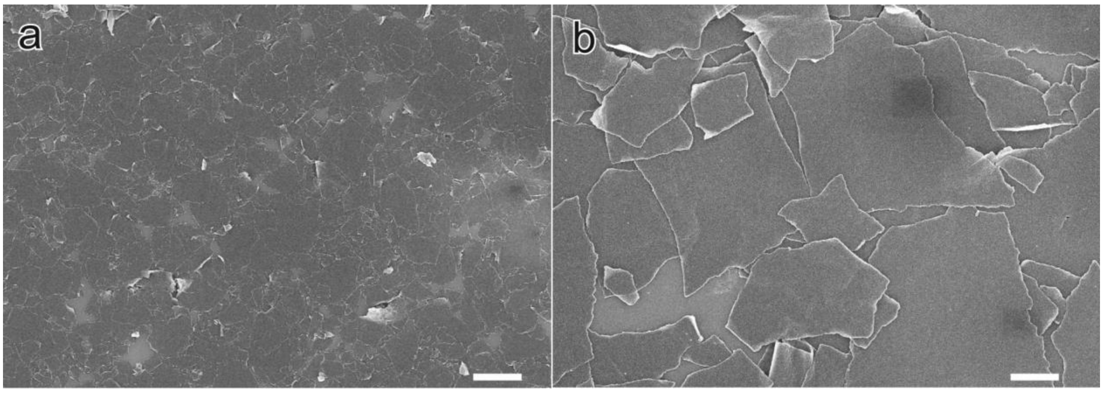

Supplementary Figure 2. SEM images of G@HMCN. Scale bars, 5 μm (**a**); 1 μm (**b**).

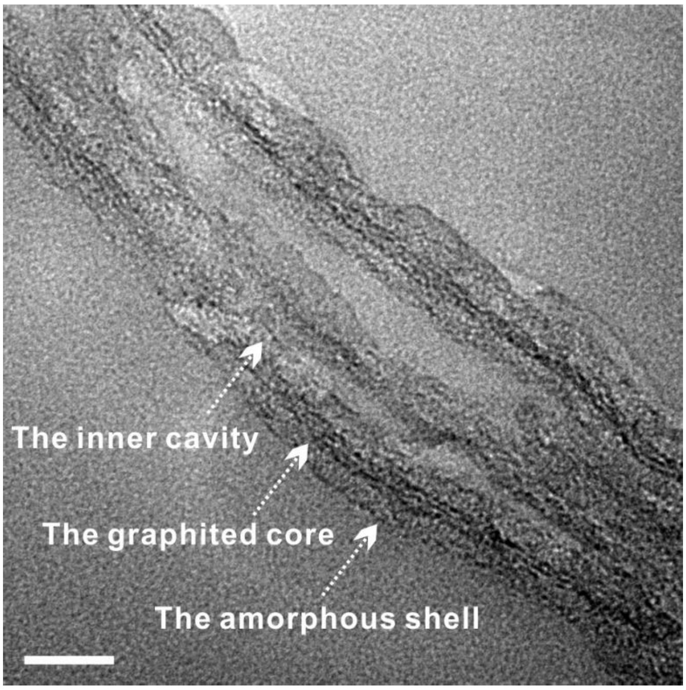

Supplementary Figure 3. Ultrathin-section TEM image of G@HMCN. Scale bar, 50 nm.

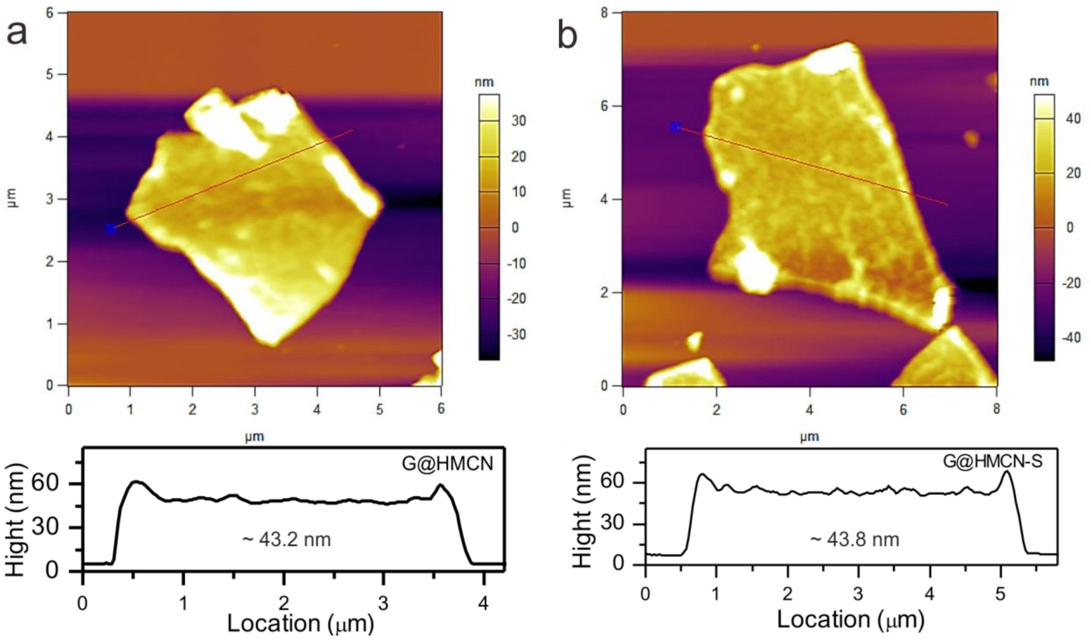

Supplementary Figure 4. Representative AFM images of G@HMCN and G@HMCN/S.

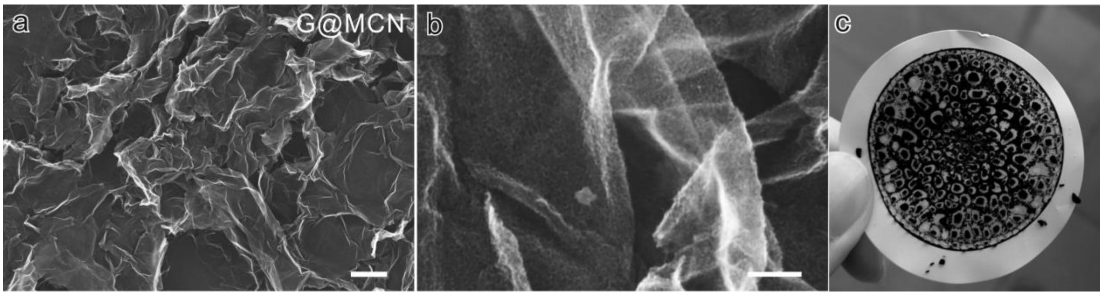

Supplementary Figure 5. Characterizations of the PB-based G@MCN core-shell nanosheets. (**a, b**) SEM images, (**c**) the PB-based G@MCN particles obtained after filtration. Scale bars, 1 μm (**a**); 100 nm (**b**).

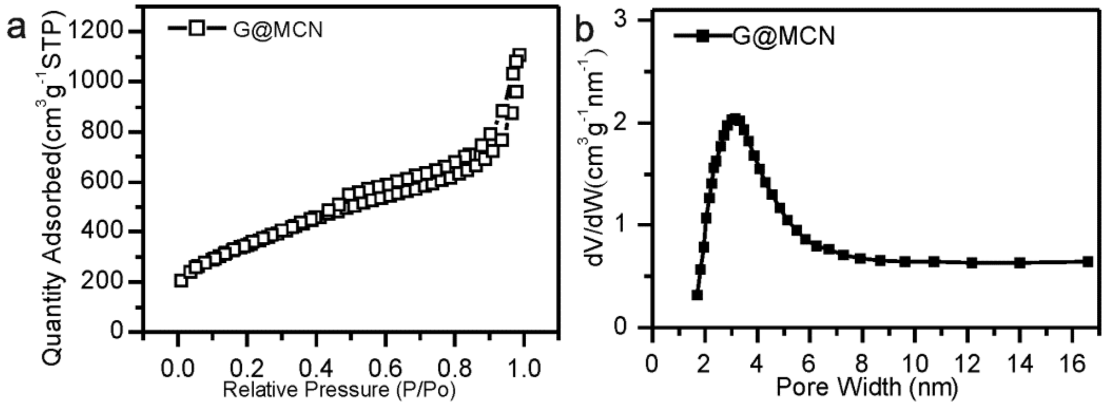

Supplementary Figure 6. The porosity of PB-based G@MCN. (**a**) N_2 sorption isotherm and (**b**) pore-size distribution of PB-based G@MCN. The BET surface area and total pore volume of PB-based G@MCN were 1387 m^2 g^{-1} and 1.43 cm^3 g^{-1}, respectively.

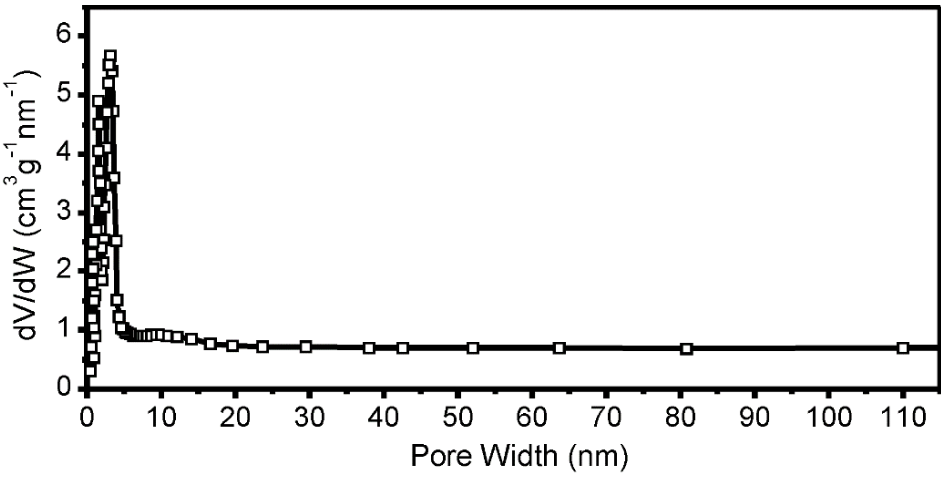

Supplementary Figure 7. The pore-size distribution of G@HMCN.

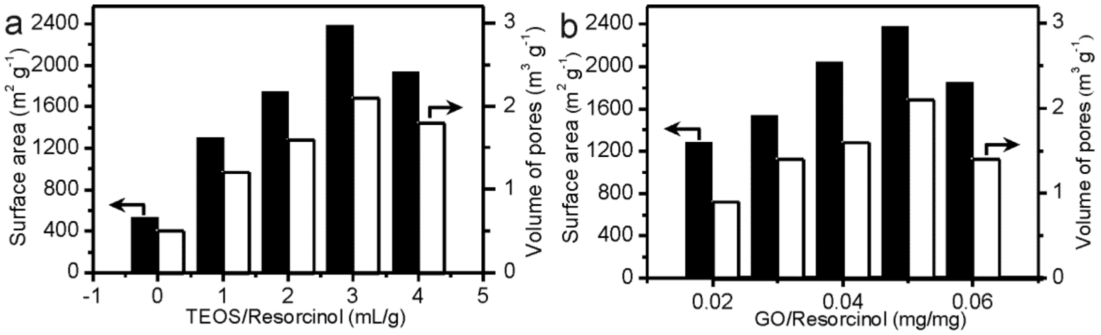

Supplementary Figure 8. The structural parameters of the different G@HMCN. (**a**) G@HMCN obtained by only adjusting the ratio of TEOS to PB precursors in the co-assembly of SiO_2 and PB process, (**b**) G@HMCN obtained by only adjusting the ratio of GO template to PB precursors.

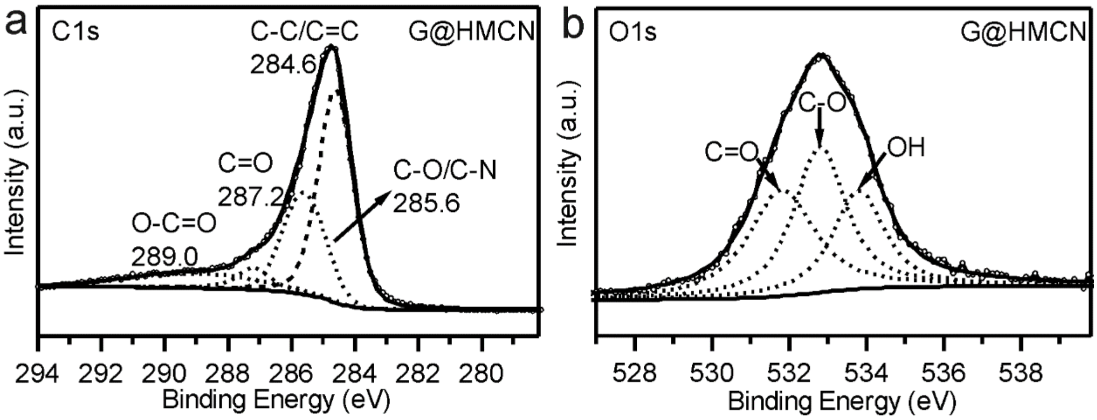

Supplementary Figure 9. XPS analysis of G@HMCN. (**a**) C 1s spectrum, (**b**) O 1s spectrum.

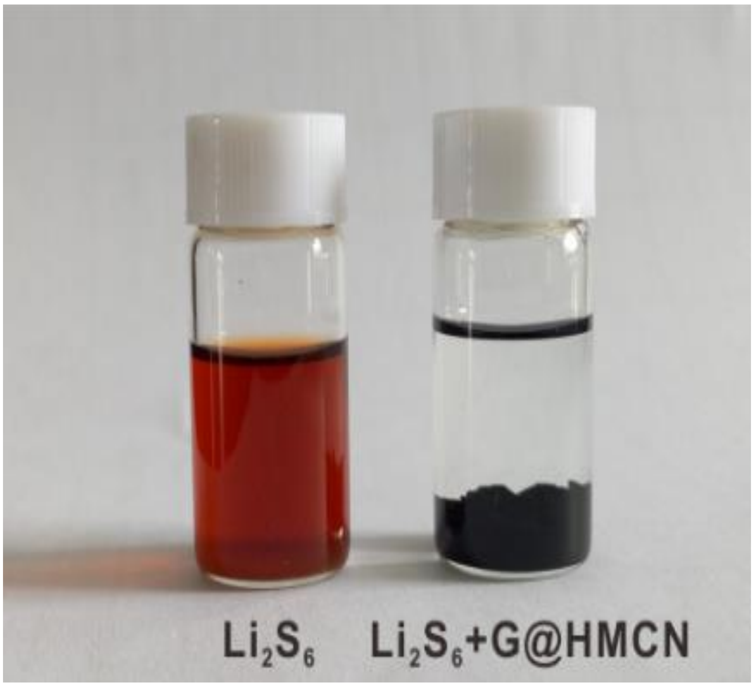

Supplementary Figure 10. Polysulfide solution treated with G@HMCN. Li_2S_6 solution was synthesized by adding Li_2S and S (molar ratio: 5:1) into tetrahydrofuran with stirring under Ar atmosphere. 100 mg of the G@HMCN was then added into the polysulfide solution containing 0.1 mmoL of Li_2S_6, followed by stirring for 15 min.

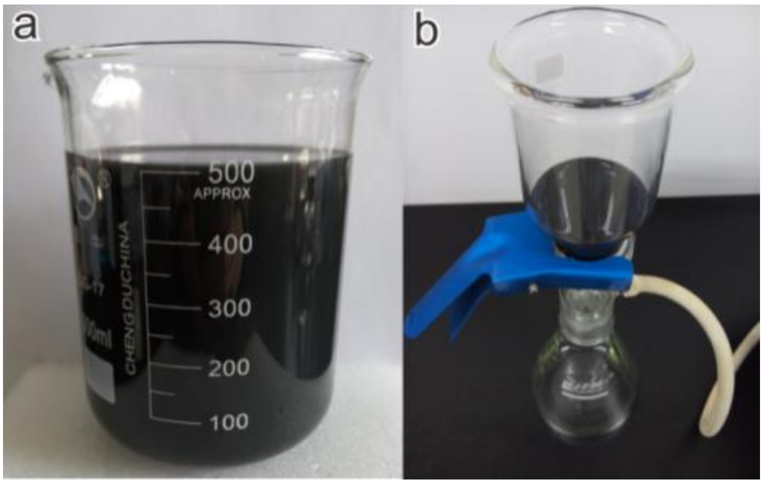

Supplementary Figure 11. Photographs for the preparation process of the G@HMCN/S-G hybrid paper.

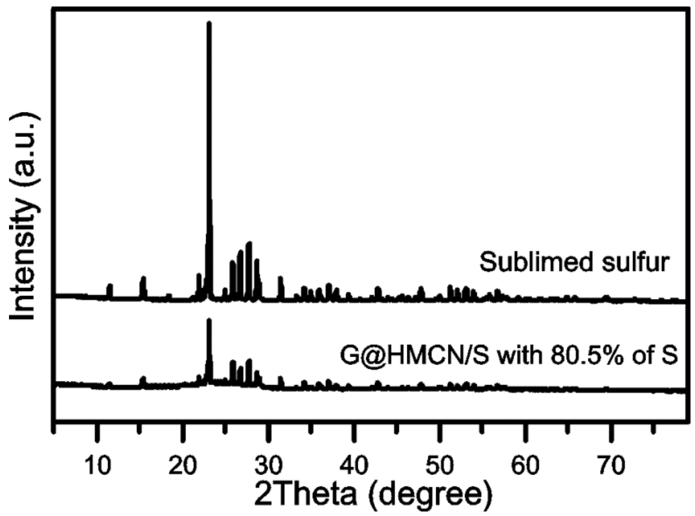

Supplementary Figure 12. XRD patterns of sublimed sulfur and G@HMCN/S.

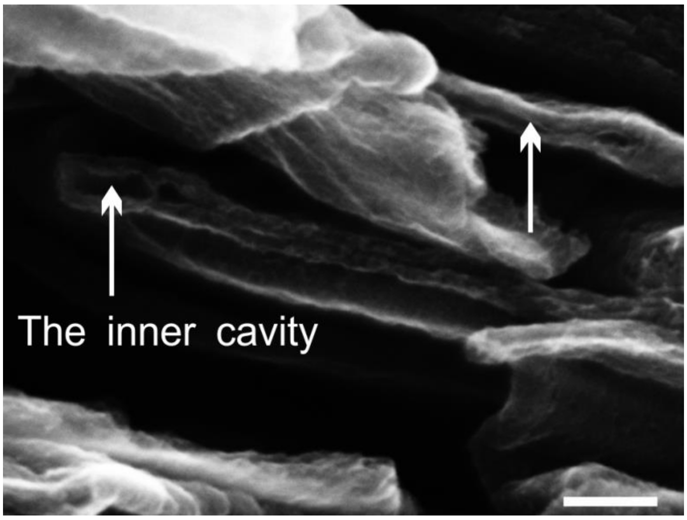

Supplementary Figure 13. Typical cross-section SEM image of the G@HMCN/S paper obtained by vacuum filtration of the G@HMCN/S aqueous dispersion. Scale bar, 100 nm.

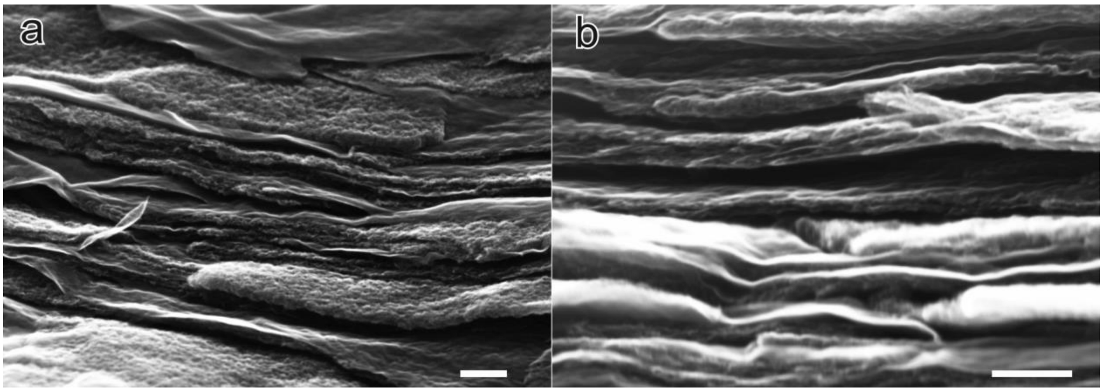

Supplementary Figure 14. The cross-section SEM images of G@HMCN/S-G. Scale bars, 100 nm.

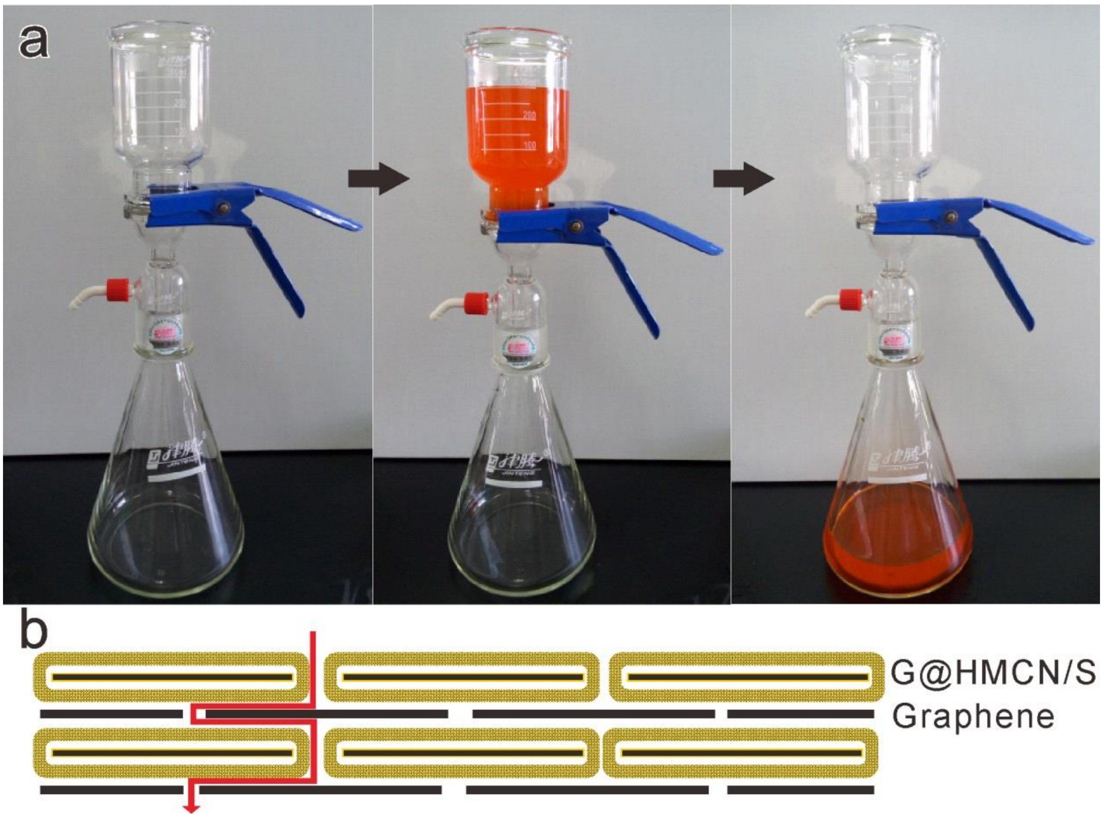

Supplementary Figure 15. The permeability of G@HMCN/S-G verified by vacuum filtration of the methyl orange solution. (a) The optical images for the vacuum filtration process, (b) a possible pathway for the mass transfer behavior of the G@HMCN/S-G paper.

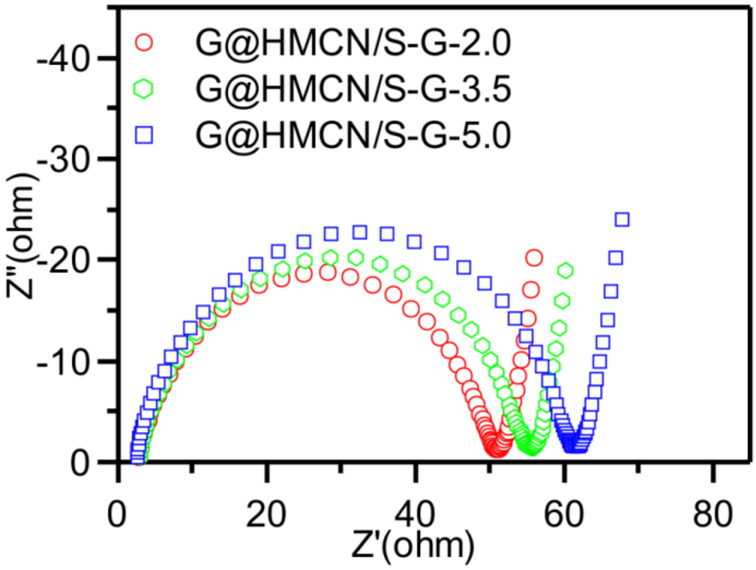

Supplementary Figure 16. Nyquist plots of the G@HMCN/S-G-2.0, G@HMCN/S-G-3.5, and G@HMCN/S-G-5.0.

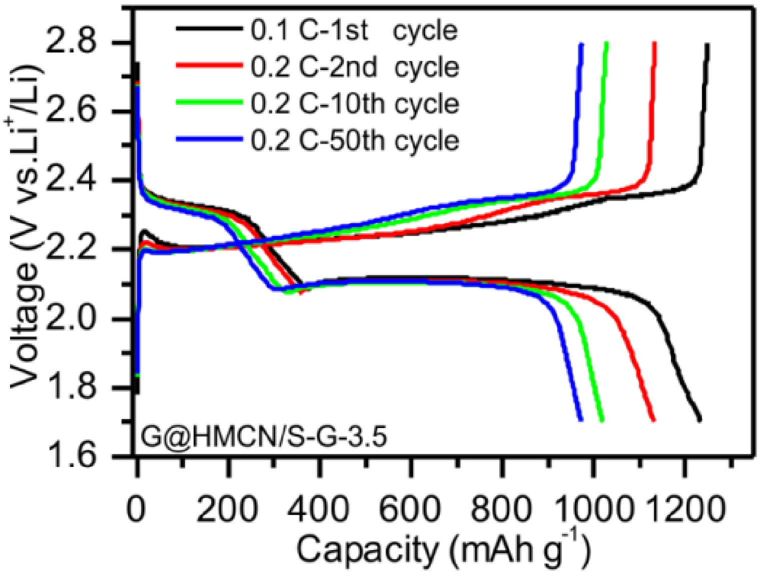

Supplementary Figure 17. Charge/discharge curves of G@HMCN/S-G-3.5 at 0.2 C.

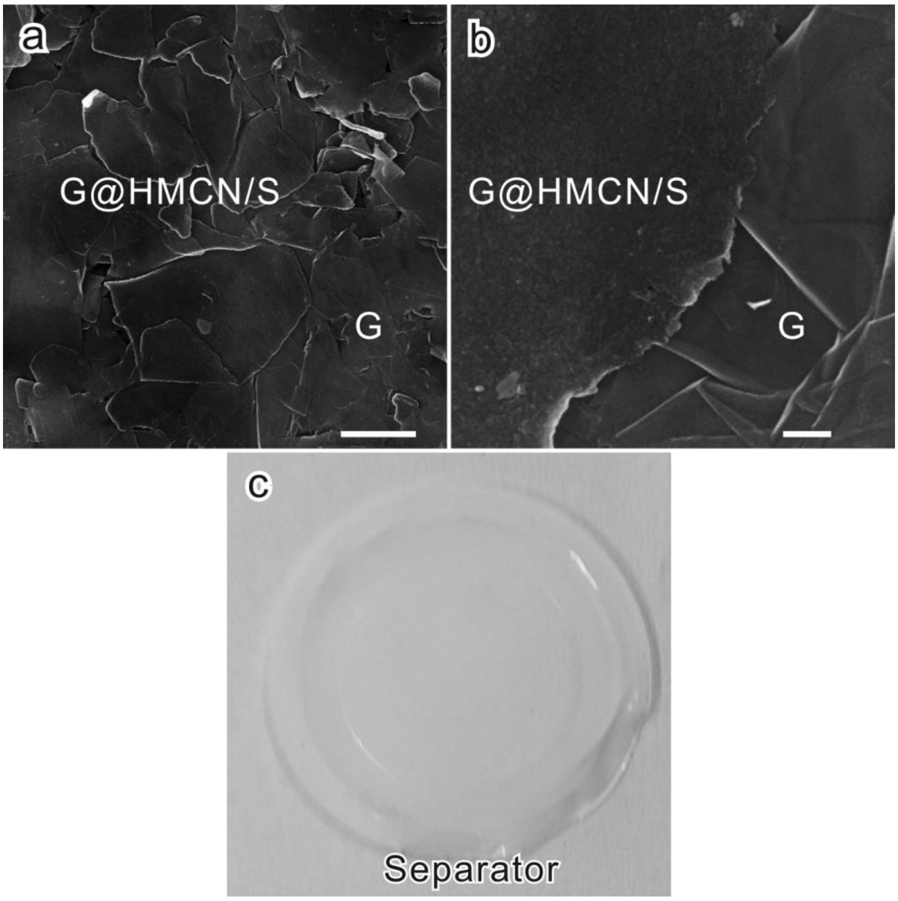

Supplementary Figure 18. Characterizations of the G@HMCN/S-5.0 coin cell after 50 cycles at 0.2 C. (a, b) SEM images of the G@HMCN/S-5.0 cathode, (c) Photos of the separator used in the G@HMCN/S-5.0 coin cell after 50 cycles at 0.2 C. The coin cell was disassembled after full charge, and the separator was directly photographed without any treatment. The top side of the separator was toward the G@HMCN/S-5.0 cathode. Scale bars, 1 μm (**a**); 100 nm (**b**).

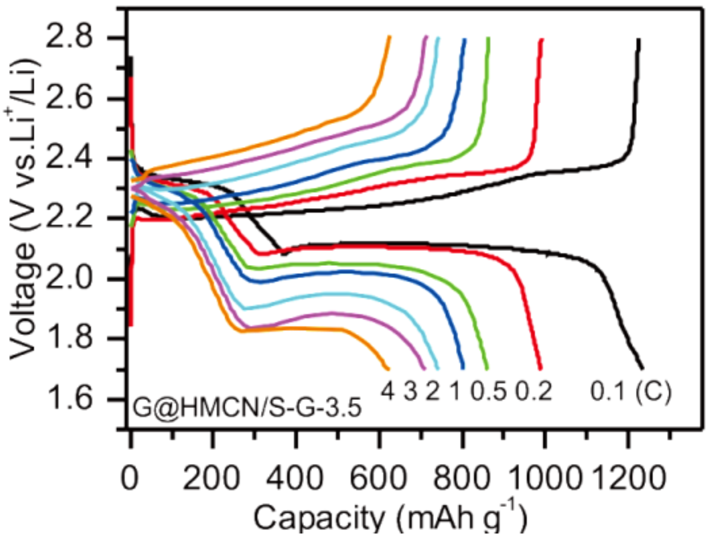

Supplementary Figure 19. Charge/discharge curves of G@HMCN/S-G-3.5 at various rates.

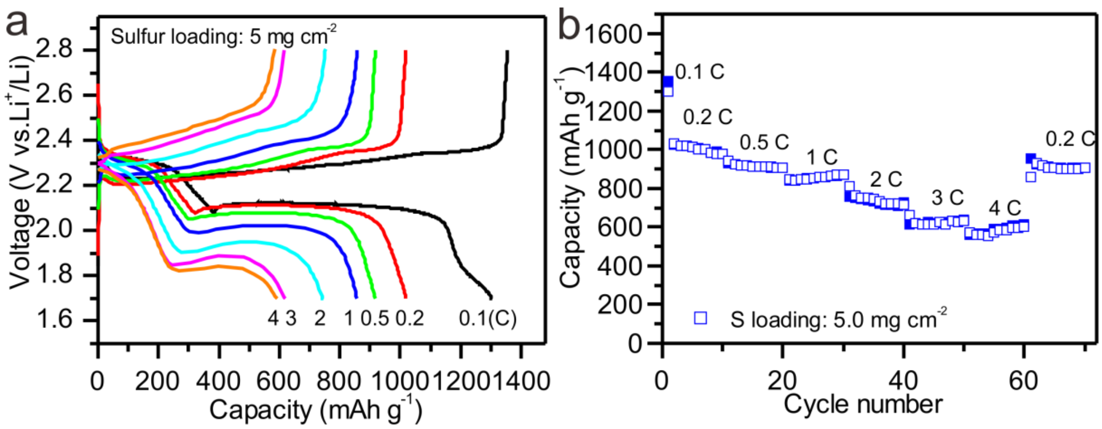

Supplementary Figure 20. The rate capability of G@HMCN/S-G-5.0 carried out from 0.2 C to 4 C after the activation at 0.1 C for only one cycle.

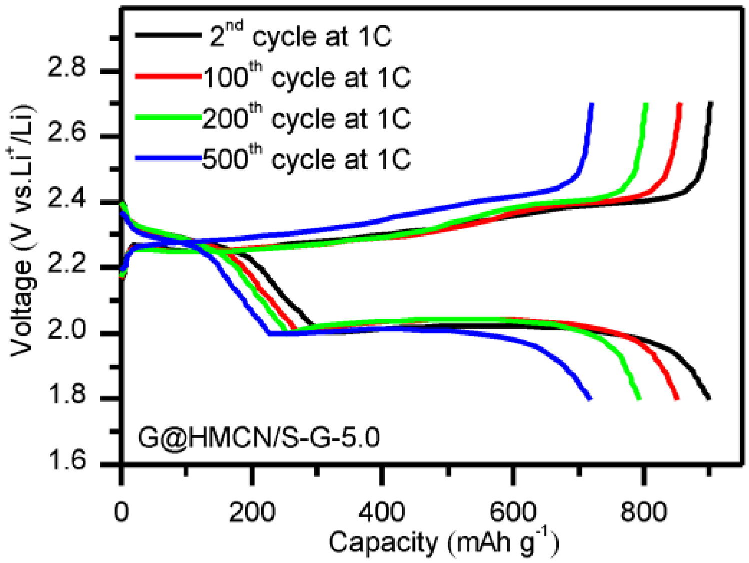

Supplementary Figure 21. Charge/discharge curves of G@HMCN/S-G-5.0 at 1 C.

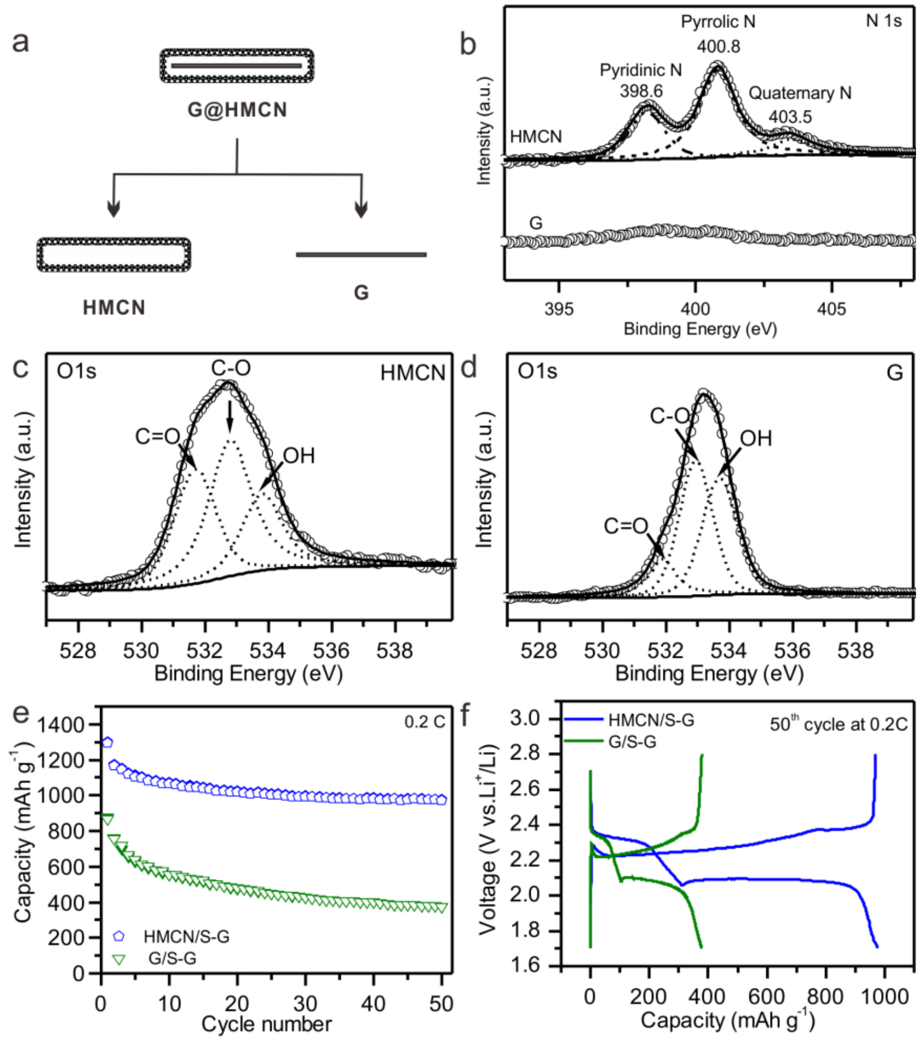

Supplementary Figure 22. The comparison of the HMCN layer and G core in the G@HMCN. (a) The structural decomposition of G@HMCN, (b) N 1s spectra of HMCN and G, (c) O 1s spectrum of HMCN, (d) O 1s spectrum of G, (e) cycling performances of HMCN/S-G and G/S-G at 0.2 C, (f) charge/discharge curves of HMCN/S-G and G/S-G after 50 cycles at 0.2 C. The G core was obtained by the carbonization of GO@SiO_2 under N_2 atmosphere at 800 ^oC for 4 h, and followed by washing with 10% HF aqueous solution for 24 h. The HMCN shell was synthesized according to the same process for G@HMCN, excepting the replacement of the GO@SiO_2 nanosheets with the SiO_2 nanosheets. The SiO_2 nanosheets were obtained by the calcination of GO@SiO_2 under air atmosphere at 500 ^oC for 5 h. The carbon/sulfur composites (i.e., HMCN/S and G/S) and carbon/sulfur cathodes (i.e., HMCN/S-G and G/S-G) were papered by the same process for G@HMCN/S-G. The loading mass of sulfur in the cathodes was 2.0 mg cm^{-2}.

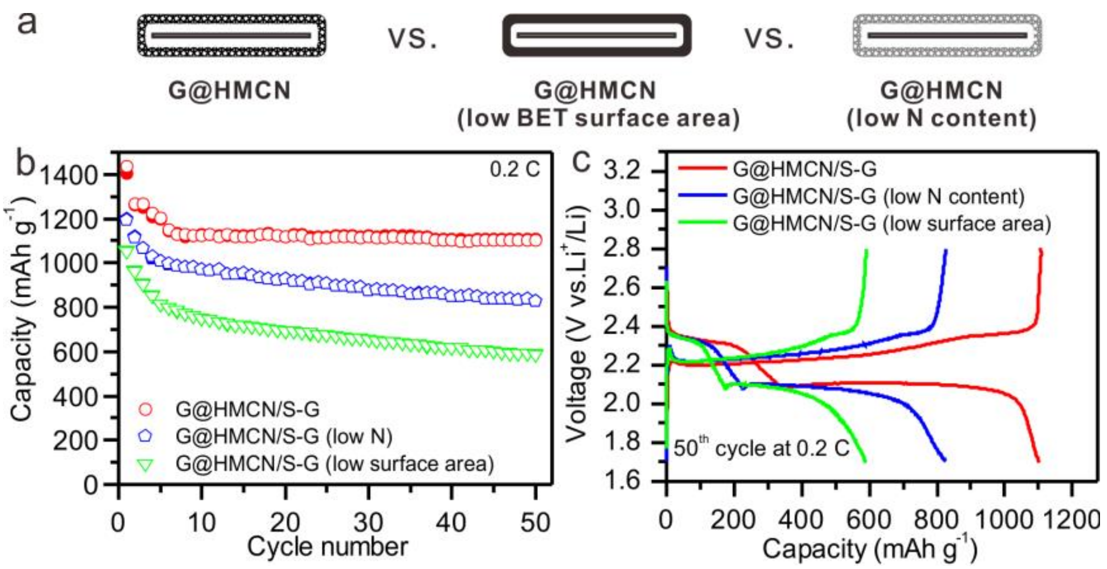

Supplementary Figure 23. The electrochemical performance of G@HMCN influenced by the surface area and N content. (a) Schematic illustration for the reference G@HMCN samples (i.e., G@HMCN with low BET surface area and G@HMCN with low N content), (c) cycling performances of the three G@HMCN/S-G samples at 0.2 C, (d) charge/discharge curves of the three G@HMCN/S-G samples after 50 cycles at 0.2 C. The loading mass of sulfur in the cathodes was 2.0 mg cm^{-2}.

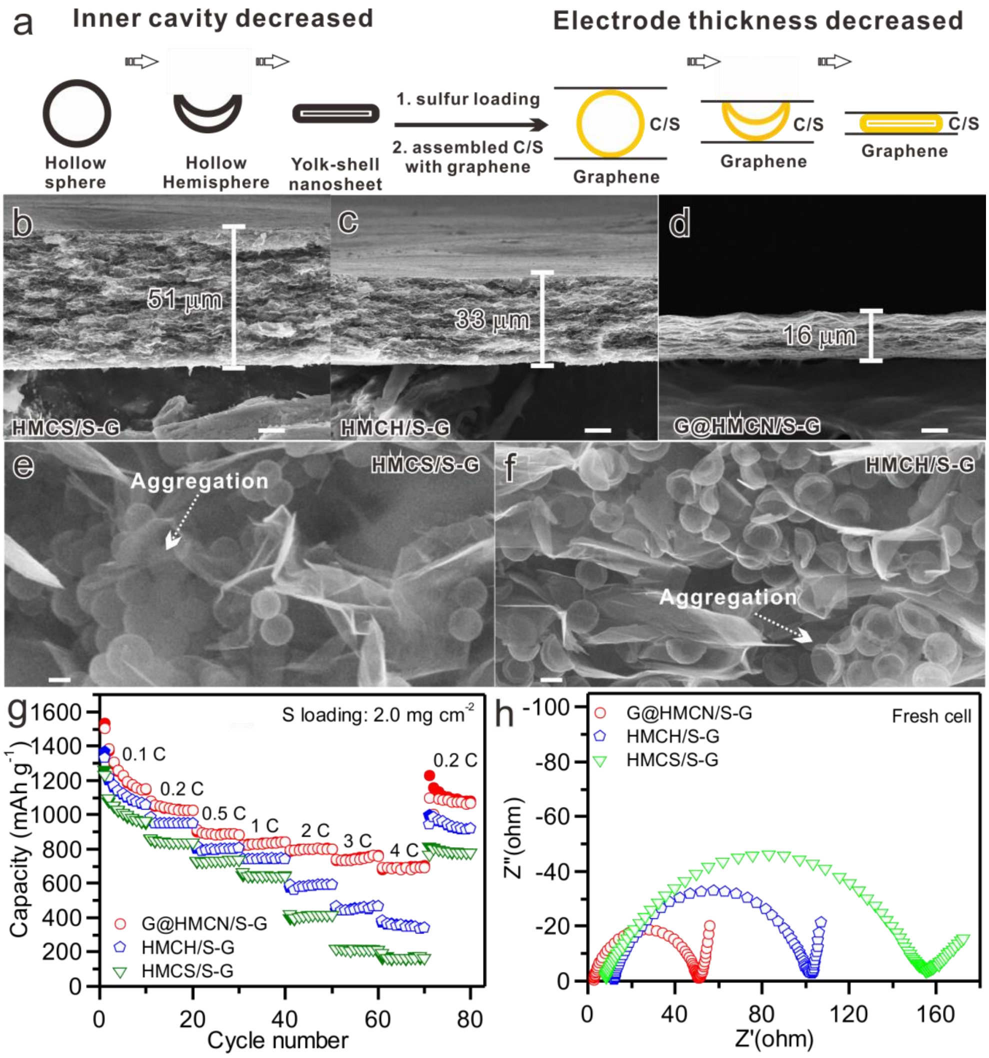

Supplementary Figure 24. The electrochemical performance of G@HMCN influenced by the inner cavity and geometric structure. (a) Schematic illustration for the structural advantage of G@HMCN in the control of the cathode thickness compared with hollow mesoporous carbon spheres (HMCS) and hollow mesoporous carbon hemispheres (HMCH), (b-f) the cross-section SEM images of HMCS/S-G (b, e), HMCH/S-G (c, f), and G@HMCN/S-G (d), (g, h) rate capabilities (g) and Nyquist plots (h) of HMCS/S-G, HMCH/S-G, and G@HMCN/S-G. The HMCS/S-G and HMCH/S-G cathodes were synthesized according to the same process for G@HMCN/S-G. The loading mass of sulfur in the three cathodes was 2.0 mg cm^{-2}. The synthesis of HMCS and HMCH was according to the reference (*Adv. Energy Mater*. **6**, 1502539, (**2016**).), and the BET surface area and N content of HMCS (1137 m^2 g^{-1}, 4.1 at%) are lower than those of HMCH (2117 m^2 g^{-1}, 5.1 at%). Scale bars, 10 μm (**b**,**c**,**d**); 200 nm (**e**,**f**).

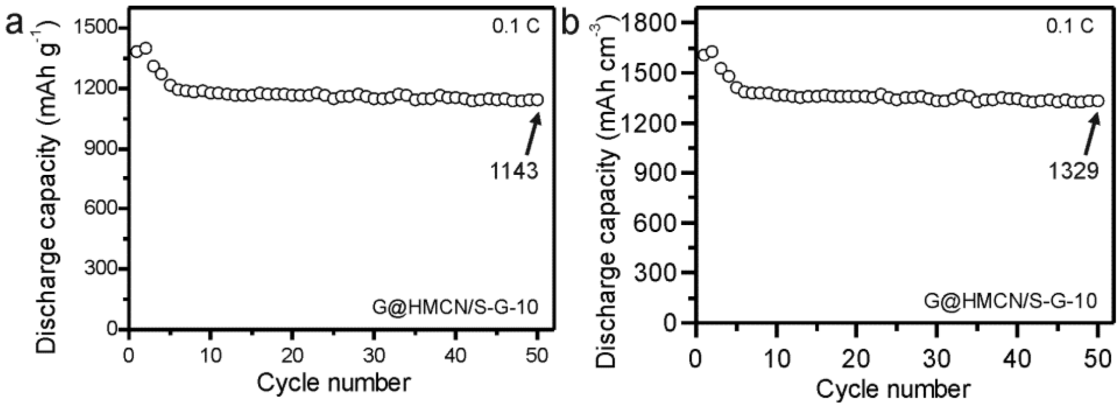

Supplementary Figure 25. Discharge capacities of G@HMCN/S-G-10 at 0.1 C.

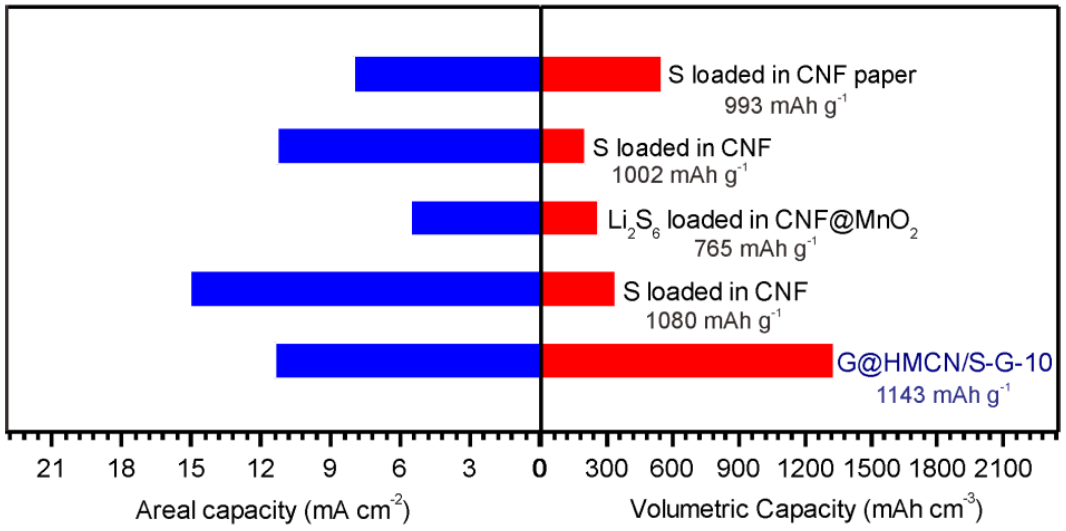

Supplementary Figure 26. The balance between the areal and volumetric capacities. Comparison of G@HMCN/S-G-10 and some state-of-the-art cathodes, including S loaded in carbon nanofiber (CNF) paper with sulfur loading of 10.8 mg cm^{-2} (*Nat. Commun.* **6**, 8850 (2015)), S loaded in CNF with sulfur loading of 11.4 mg cm^{-2} *(Adv. Mater.* **27**, 1694 (2015)), Li_2S_6 loaded in CNF@MnO_2 with sulfur loading of 7.2 mg cm^{-2} *(Nano Energy* **26**, 224 (2016)), and S loaded in CNF with sulfur loading of 18.9 mg cm^{-2} *(Adv. Energy Mater.* **6**, 1502459 (2016)). The inset specific capacities reveal a higher sulfur utilization of G@HMCN/S-G-10 at high sulfur loading and high sulfur content.

Supplementary Table 1. The rate properties of the high-sulfur-loading cathodes (>2 mg cm^{-2}).

Sulfur cathodes	S loading (mg cm^{-2})	S content (wt%)[a]	The maximal rate (C)[b]	Capacity (mAh g^{-1})	Ref
C/S composites encapsulated in nickel hydroxides*	~2.5	62.4	4<br>5	289<br>195	1
S loaded in MnO_2@hollow CNF*[c]	3.5	49.7	1	690	2
S loaded in $Co(OH)_2$@LDH Nanocages*	3.0	52.5	1	500	3
S loaded in TiO@C	4.0	49	0.2	630	4
S loaded in N-doped graphene sheets*	5.0	64	1	650	5
S loaded in CNF paper**	3.6	72.3	2	363	6.
Li_2S_6 loaded in 3D N/S-doped graphene**	4.6	63~72.5	2	430	7
S loaded in hollow Carbon spheres and graphene**	3.9	62	3	430	8
S loaded in CNF**	5.7	52.9	2	300	9
S loaded in porous CNF**	4.5	72	2	<300	10
S loaded in N-doped CNT-graphene hybrid**[d]	4.2	58.4	2<br>5	532<br>273	11
Li_2S_6 loaded in CNF@MnO_2**	4.8	~71	1	627	12
**G@HMCN/S-G-5.0**[e]**	**5.0**	**73**	**4**	**524**	**This work**

*The slurry-based cathodes,**the self-supporting cathodes, (a) the S content is calculated based on the cathode, (b) 1C = 1675 mA g^{-1}, (c) CNF = cabron nanofibers, (d) CNT = carbon nanotubes, (e) the discharge capacities of G@HMCN/S-G-5.0 at 1, 2, and 3 C were 733, 698, and 593 mAh g^{-1}, respectively.

Supplementary Table 2. The rate properties of the low-sulfur-loading cathodes (<2 mg cm^{-2}).

Sulfur cathodes	S content (wt%)	S loading (mg cm^{-2})	Rate (C)*	Capacity (mAh g^{-1})	Ref
S loaded in TiN	51	1.0	0.1~1	1121~776	13
S loaded in carbon nanobowls	49	1.1-1.5	0.2~2~4	1065~673~535	14
S loaded in carbon nanosheets	51.2	1~1.5	0.1~2	1250~738	15
S loaded in Co-C-N	49	2.0	0.2~2~5	1145~685~565	16
S loaded in carbon microrods	71	1.5	0.5~2~5	783~620~320	17
S loaded in N/B-doped carbon	56	0.9~1.2	0.2~2~3	1050~555~480	18
S loaded in GO-nc-Fe	47~55	1.0	0.2~2	1179~724	19
S loaded in NH_2 modified CNT	56	1.2	0.5~2~4	975~690~300	20
S loaded in oxygenated CN	39.2	<2.0	0.1~1	922~517	21
S loaded in hollow carbon spheres	63	2.0	0.1~3	1200~733	22
**G@HMCN/S-G-2.0**	**73**	**2.0**	**0.1~2~4**	**1224~801~690**	**This work**

Supplementary Table 3. Cyclabilities of the high-sulfur-loading cathodes (>2 mg cm^{-2}).

Sulfur cathodes	S loading (mg cm^{-2})	Capacity (mAh g^{-1})	Ref
S loaded in MnO_2@hollow CNF	3.5	662 (300th cycle at 0.5 C)	2
S loaded in $Co(OH)_2$@LDH Nanocages	3.0	491 (100th cycle at 0.5 C)	3
Li_2S_6 loaded in 3D N/S-doped graphene	4.6	550 (500th cycle at 0.5 C)	7
S loaded in hollow Carbon spheres and graphene	3.9	520 (200th cycle at 0.2 C)	8
S loaded in CNF	5.7	~735 (200th cycle at 0.2 C).	9
S loaded in porous CNF	4.5	680 (200th cycle at 0.2 C)	10
S loaded in C_3N_4 Nanosheets	3.0	~600 (175th cycle at 0.2 C)	23
**G@HMCN/S-G-5.0**	**5.0**	**719 (500th cycle at 1 C)**	**This work**

Supplementary Table 4. The thickness of the high-sulfur-loading cathodes (>2 mg cm^{-2}).

**Sulfur cathodes**	**S loading (mg cm^{-2})**	**Thickenss (μm)**	**S/thickness (mg cm^{-2} μm^{-1})**	**Ref.**
S loaded in $Co(OH)_2$@LDH Nanocages*	3.0	`~45	~0.067	3
S loaded in CNF paper	3.6	~60	~0.06	6
S loaded in hollow Carbon spheres and graphene	3.9	~120	~0.033	8
S loaded in CNF	11.4	380	0.03	9
S loaded in porous CNF	4.5	>100	<0.045	10
**G@HMCN/S-G-5.0**	**5.0**	**43**	**0.12**	**This work**

Supplementary Table 5. Capacities of the high-sulfur-loading cathodes (>10 mg cm^{-2}).

**Sulfur cathodes**	**S loading (mg cm^{-2})**	**Cathode area (cm^{-2})**	**S content (wt%)**	**Capacity (mAh g^{-1})**	**Ref**
S loaded in carbon nanofiber paper	10.8	~1	72.3	760 (50th cycle at 0.066 C)	6
S loaded in cabron nanofibers	11.4	0.785	56.3	650 (100th cycle at 0.2 C)	9
S loaded in carbon cotton	30.7<br>61.4	0.25<br>0.25	80<br>80	890 (50th cycle at 0.1 C)<br>724 (50th cycle at 0.1 C)	24
S loaded in the core-shell cathode	10	1	58.1	~800 (50th cycle at 0.1 C)	25
CNT/S loaded in 3D Al foam	12.5	1.33	42.5	250 (70th cycle at 0.1 C)	26
S loaded in 3D carbon nonwoven	15.6	0.785	58.4	700 at 0.025 C*<br><500 at 0.1 C*	27
**G@HMCN/S-G-10**	**10**	**1.13**	**73**	**1140 (50th cycle at 0.1 C)**	**This work**

***** The capacities are obtained from the rate capability test.

Supplementary Table 6. The volumetric capacities of the self-supporting carbon/sulfur cathodes with high sulfur loading (>2 mg cm^{-2}).

**Sulfur cathodes**	**Capacity ($mAh\ cm^{-3}$)**	**Ref**
S loaded in CNF paper	549 (2nd cycle at 0.2 C)	6
S loaded in CNF	~200 (100th cycle at 0.2 C)	9
Li_2S_6 loaded in CNF@MnO_2	262 (1st cycle at 0.2 C)	12
S loaded in CNF	335 (1st cycle at 0.05 C)	28
**G@HMCN/S-G-5.0**	**1050 (50th cycle at 0.2 C)**	**This work**
**G@HMCN/S-G-10**	**1329 (50th cycle at 0.1 C)**	**This work**

Supplementary References

1. Jiang, J*, et al.* Encapsulation of sulfur with thin-layered nickel-based hydroxides for long-cyclic lithium-sulfur cells. *Nat. Commun.* **6,** 8622 (2015).
2. Li, Z., Zhang, J. T. & Lou, X. W. Hollow carbon nanofibers filled with MnO_2 nanosheets as efficient sulfur hosts for lithium-sulfur batteries. *Angew. Chem. Int. Ed.* **54,** 12886–12890 (2015).
3. Zhang, J. T., Hu, H., Li, Z. & Lou, X. W. Double-shelled nanocages with cobalt hydroxide inner shell and layered double hydroxides outer shell as high-efficiency polysulfide mediator for lithium-sulfur batteries. *Angew. Chem. Int. Ed.* **55,** 3982–3986 (2016).
4. Li, Z., Zhang, J., Guan, B., Wang, D., Liu, L. M. & Lou, X. W. D. (2016). A sulfur host based on titanium monoxide@carbon hollow spheres for advanced lithium–sulfur batteries. *Nat. Commun.* **7,** 13065 (2016).
5. Song, J., Yu, Z., Gordin, M. L. & Wang, D. (2016). Advanced sulfur cathode enabled by highly crumpled nitrogen-doped graphene sheets for high-energy-density lithium-sulfur batteries. *Nano Lett.* **16,** 864–870 (2016).
6. Li, Z., Zhang, J. T., Chen, Y. M., Li, J. & Lou, X. W. Pie-like electrode design for high-energy density lithium-sulfur batteries. *Nat. Commun.* **6,** 8850 (2015).
7. Zhou, G. M., Paek, E., Hwang, G. S. & Manthiram, A. Long-life Li/polysulphide batteries with high sulphur loading enabled by lightweight three-dimensional nitrogen/sulphur-codoped graphene sponge. *Nat. Commun.* **6,** 7760 (2015).
8. Zhou, G., Zhao, Y. & Manthiram, A. Dual-confined flexible sulfur cathodes encapsulated in nitrogen-doped double-shelled hollow carbon spheres and wrapped with graphene for Li-S batteries. *Adv. Energy Mater.* **5,** 1402263 (2015).

9. Qie, L. & Manthiram, A. A facile layer-by-layer approach for high-areal-capacity sulfur cathodes. *Adv. Mater.* **27,** 1694–1700 (2015).
10. Zhou, W. D., Guo, B. K., Gao, H. C. & Goodenough, J. B. Low-cost higher loading of a sulfur cathode. *Adv. Energy Mater.* **6,** 1502059 (2016).
11. Ding, Y. L., *et al.* Solid-State Growth of 3D Well-Interconnected Nitrogen-Rich Carbon Nanotube-Graphene Hybrid Architectures for Lithium-Sulfur Batteries. *Adv. Funct. Mater.* **26,** 1112–1119 (2016).
12. Xu, H. Qie, L., & Manthiram, A. An integrally-designed, flexible polysulfide host for high-performance lithium-sulfur batteries with stabilized lithium-metal anode. *Nano Energy* **26,** 224–232 (2016).
13. Cui, Z. Zu, C., Zhou, W., Manthiram, A. & Goodenough, J. B. Mesoporous titanium nitride-enabled highly stable lithium-sulfur batteries. *Adv. Mater.* **28,** 6926–6931 (2016).
14. Pei, F. *et al.* From hollow carbon spheres to N-doped hollow porous carbon bowls: rational design of hollow carbon host for Li-S batteries. *Adv. Energy Mater.* **6,** 1502539 (2016).
15. Rehman, S. *et al*. 3D vertically aligned and interconnected porous carbon nanosheets as sulfur immobilizers for high performance lithium-sulfur batteries. *Adv. Energy Mater.* **6,** 1502518 (2016).
16. Li, Y. J., Fan, J. M., Zheng, M. S. & Dong, Q. F. A novel synergistic composite with multi-functional effects for high-performance Li-S batteries. *Energy Environ. Sci.* **9,** 1998–2004 (2016).
17. Zheng, Z. M. et al. High sulfur loading in hierarchical porous carbon rods constructed by vertically oriented porous graphene-like nanosheets for Li-S batteries. *Adv. Funct. Mater.* **26,** 8952–8959 (2016).
18. Yuan, S. Y. *et al.* Graphene-supported nitrogen and boron rich carbon layer for improved performance of lithium-sulfur batteries due to enhanced chemisorption of lithium polysulfides. *Adv. Energy Mater.* **6,** 151733 (2016).

19. Mi, Y. *et al.* ferrocene-promoted long-cycle lithium-sulfur batteries. *Angew. Chem. Int. Ed.* **128,** 15038–15042 (2016).
20. Ma, L. *et al.* Enhanced Li-S batteries using amine-functionalized carbon nanotubes in the cathode. *ACS Nano* **10,** 1050–1059 (2016).
21. Liu, J. *et al.* A graphene-like oxygenated carbon nitride material for improved cycle-life lithium/sulfur batteries. *Nano letters* **15,** 5137–5142 (2015).
22. Li, W. *et al.* A sulfur cathode with pomegranate-like cluster structure. *Adv. Energy Mater.* **5,** 1500211 (2015).
23. Pang, Q. & Nazar, L. F. Long-life and high-areal-capacity Li–S batteries enabled by a light-weight polar host with intrinsic polysulfide adsorption. *ACS nano* **10,** 4111–4118 (2016).
24. Chung, S. H., Chang, C. H. & Manthiram, A. A carbon-cotton cathode with ultrahigh-loading capability for statically and dynamically stable lithium-sulfur batteries. *ACS Nano* **10,** 10462–10470 (2016).
25. Chung, S. H., Chang, C. H. & Manthiram, A. A core-shell electrode for dynamically and statically stable Li-S battery chemistry. *Energy Environ. Sci.* **9,** 3188–3200 (2016).
26. Cheng, X. B. *et al.* Three-dimensional aluminum foam/carbon nanotube scaffolds as long-and short-range electron pathways with improved sulfur loading for high energy density lithium-sulfur batteries. *J. Power Sour.* **261,** 264–270 (2014).
27. Hagen, M. *et al.* Development and costs calculation of lithium-sulfur cells with high sulfur load and binder free electrodes. *J. Power Sour.* **224,** 260–268 (2013).
28. Qie, L., Zu, C. & Manthiram, A. A high energy lithium-sulfur battery with ultrahigh-loading lithium polysulfide cathode and its failure mechanism. *Adv. Energy Mater.* **6,** 1502459 (2016).
